# The influence of the trainer on the motivation and resilience of sportspeople: A study from the perspective of self-determination theory

**DOI:** 10.1371/journal.pone.0221461

**Published:** 2019-08-20

**Authors:** Rubén Trigueros, José Manuel Aguilar-Parra, Adolfo J. Cangas-Díaz, José M. Fernández-Batanero, Miguel A. Mañas, Víctor B. Arias, Remedios López-Liria

**Affiliations:** 1 Faculty of Languages and Education, University of Antonio de Nebrija, Madrid, Spain; 2 Faculty of Education Science, Department of Psychology, University of Almería, Almería, Spain; 3 Faculty of Psychology, Department of Psychology, University of Almería, Almería, Spain; 4 Faculty of Education Science, Teaching and Educational Organization, University of Seville, Seville, Spain; 5 Faculty of Psychology, Personality, Assessment and Psychological Treatment University of Salamanca, Salamanca, Spain; 6 Faculty of Health Science, Department of Nursing Science, Physiotherapy and Medicine, Health Research Centre, University of Almería, Almería, Spain; University of Zaragoza, SPAIN

## Abstract

The aim of this study was to evaluate the influence of a trainer’s interpersonal relations from the perspective of autonomy support and controlling style on sportspeople’s basic need satisfaction and frustration, motivation, and resilience. The study used a cross-sectional design based on self-determination theory (SDT). Sportspeople (*N* = 324) completed questionnaires to measure their perceptions of trainers’ autonomy-supportive and controlling coaching styles, basic need satisfaction and frustration in the sports context, motivation for sport, and resilience. Structural equation modeling of the proposed relations among variables supported SDT by showing a positive relation between perceived autonomy support and the satisfaction of basic psychological needs (β = .39, *p* < .001) and a negative relation with the frustration of psychological needs (β = −.17, *p* < .05). The coach’s perceived interpersonal controlling style showed a positive relation with the frustration of psychological needs (β = .55, *p* < .001) and a negative relation with the satisfaction of basic psychological needs (β = −.27, *p* < .05). Furthermore, autonomous motivation showed a negative relation (β = −.46, *p* < .001) with the frustration of psychological needs and a positive relation (β = .35, *p* < .05) with the satisfaction of basic psychological needs and resilience (β = .60, *p* < .001). In addition, the resilience of sportspeople was indirectly affected to the same extent by the trainer’s influence through control (β = −.38, *p* < .05) and perception of autonomy support (β = .16, *p* < .05) through the mediators of satisfaction of basic psychological needs and motivation. These results show the influence of the coach on the motivation and resilience of sportspeople.

## Introduction

Among the main internal goals of sportspeople, we can enumerate the consolidation of healthy habits, awareness of the benefits of physical activity through sport, encouragement of physical, social, and psychic development, and increased efficiency in reaching their set goals through the improvement of their abilities [1]. Thus, such authors as Vella et al. [2] describe the trainer as having a possibly significant influence on sportspeople’s activities and the development of their personalities and cognitive capacities based on their degree of interaction. Thus, the trainer should understand the importance of every practice in sport preparation, paying attention to the transformation and personal change of every sportsperson socially (prosocial and antisocial behaviors in social situations or experience of a sense of effectiveness in interacting with one’s environment), cognitively (self-esteem and engagement in sport activities), and affectively (experiences of well-being, life satisfaction) [3]. However, there have been only a few studies of how a trainer’s interpersonal style influences sportspeople through their motivation for the sport and psychological resilience [4]. The aim of this study is to evaluate the influence of the trainer’s interpersonal relations on sportspeople’s motivation and resilience from the perspective of autonomy support and controlling style.

## Self-determination theory

Some studies in the sports field have sought to analyze the motivation of sportspeople according to the self-determination theory (SDT) approach [5–7]. This theory [8] suggests that there are three basic psychological needs: Autonomy (the extent to which individuals feel responsible for the initiation of their behaviors); competence (when individuals are able to reach their desired goals); and relatedness (when people feel integrated into a group), which have been defined as essential emotional nutrients of an individual’s development, integrity, and well-being [9,10].

Individuals inherently seek to explore opportunities to satisfy their needs, feeling energized and joyful during interactions when they are satisfied and frustrated when they are thwarted. Based on these experiences, people construct views of themselves and the world in relation to these needs and the expectations that shape their participation in their environment [11].

SDT [8] addresses the types of motivation, “paying attention to autonomous motivation, controlled motivation, and amotivation as predictors of performance, relational, and well-being outcomes.” The satisfaction of psychological needs leads to autonomous motivation, their thwarting to controlled motivation [12–14].

In addition, SDT [15,16] and more recent research [17–19] suggest that a social agent’s behaviors may be conceptualized as autonomy-support (supporting self-initiated striving and creating conditions to experience a sense of volition, choice, and self-endorsement), controlling behaviors (coercive, pressuring, and authoritarian in imposing a specific, preconceived way of thinking), competence support (or structure), competence thwarting, relatedness support, and relatedness thwarting [17–19].

Sports research based on SDT has analyzed how autonomy support is associated with the three basic psychological needs, autonomous motivation, and adaptative consequences (e.g., vitality, interest, effort, concentration, self-esteem) [20,21], and has demonstrated that autonomy-supportive coach behaviors are related to autonomous motivation [5,22], while a controlling interpersonal style can induce controlled motivation [6,16].

This study sought to determine how the trainer may influence sportspeople through two different interpersonal styles: support of autonomy and control of conduct. For example, if the trainer offers sportspeople choices during training sessions (i.e., sportspeople participate in decisions about everything involved in their physical activities), this may help them to develop an autonomous motivation (i.e., enjoyment, interest, or identification, and even an acceptance of responsibility for behavior and internalization). Sportspeople would be quite likely to experience a perceived locus of causality internal to themselves as the origin of their behaviors, experiencing internally an autonomous motivation, thereby contributing to the satisfaction of the three basic psychological needs (an authentic sense of self-direction and volition, opportunities to be effective in one’s sport, and to express one’s capacities) [23].

On the other hand, a controlling coaching style on the part of the trainer giving priority to external pressures, such as the use of coercive methods and impositions (controlling use of rewards, verbal abuse and threats, yelling, and guilt induction) and acting with preconceived ideas, may be perceived by sportspeople as the origin of their behaviors [15, 24], causing their basic psychological needs to be thwarted, or experienced as frustrating their development (i.e., humiliation and belittlement). Therefore, sportspeople could perceive a locus of causality external to themselves as the origin of their behaviors, experiencing internally a non-autonomous motivation [24].

There have been some studies in the sports field seeking to analyze the influence of the trainer on the sportsperson. The study of Moreno, Parra, and González-Cutre [25] applied a multivariate analysis of people practicing collective sports and concluded that a working environment created by the trainer in which an orientation toward physical activity and the sportsperson’s self-decision capacity prevails favors the development of the three basic psychological needs. Moreover, it provided evidence that competence, relatedness, and autonomy may affect autonomous motivation and the enjoyment of sport [25]. Later, Ramis, Torregrosa, Viladrich, and Cruz [26] used structural equation modeling to investigate the influence of peers, the trainer, and parents on a sample of 278 beginner sportspeople aged from 11 to 17. The results showed that the autonomy support of peers, parents, and trainer affected the sportspeople’s autonomous motivation positively and extrinsic motivation negatively.

In addition, there exist studies with a more complete design analyzing the influence of the trainer on the support of sportspeople’s autonomy, such as Balaguer et al. [7], who found that support of autonomy affected two of the three psychological needs (autonomy and relatedness). At the same time, the three basic psychological needs affected self-determined motivation [7]. Finally, self-determined motivation favored satisfaction with life and self-esteem. These studies [25–27] are in line with Vallerand and Mageau’s results [28], which suggest that the perception of support of autonomy by the trainer would facilitate the satisfaction of basic needs, which in turn would be related positively to autonomous motivation, with consequent positive social, affective, and cognitive effects.

Other research has analyzed the influence of the support of autonomy on the part of the trainer to examine the psychological well-being of sportspeople [29]. In particular, Rivas and García-Mas [30] applied regression analysis to 85 footballers, showing that the trainer’s support of autonomy affects the sportsperson’s psychological and general well-being in consonance with the SDT. Autonomous sportspeople will experience higher levels of positive behavior and personal fit, leading to desirable consequences such as health and psychological, physical, and emotional well-being, than will those who are more controlled [7,31].

SDT suggests that satisfaction of the basic psychological needs (BPN) improves well-being and strengthens inner resources related to resilience among both athletes [24] and sport coaches [32], whereas frustration in these three areas increases one’s vulnerability to defense mechanisms [33,34].

### Conceptualization of resilience

Resilience has been variously conceptualized in the literature. A previous review of psychological resilience considers the construct as being operationalized in variety of ways, but most definitions involve adversity (i.e., potential negative effects of stressors) and positive adaptation (behavior promoting personal assets, coping processes, and symptoms related to internal well-being) [35–37]. The main studies in this matter concern health care [38], the military [39], and the workplace [40], with few such studies of sports [27]. In a study of sport psychology, Cowden et al. (2016) [41] propose a conceptualization of resilience as “associated with the possession of and/or the presence of protective (e.g., personal, familial, community [42]) and vulnerability factors that influence the risk-positive adaptation relationship” [43] (p. 7).

Thus, this study involves a construct of resilience similar to that of Sarkar and Fletcher [44], which plays an important role in sports, since its development is linked to the sportsperson’s emotional and cognitive well-being (acceptance of one’s self). It refers to the set of personal competences that constitute the human ability to overcome unfavorable and stressful situations, as well as sustaining the positive growth of the individual as a result of the achievement of his/her own personal and sports challenges. Resilience contributes to the development of an exhaustive process of behavioral, social, and affective adaptation [44].

Thus, resilience is a highly context-dependent process that constantly changes over time [45]. Currently, research in the domain of sports holds that resilience is a dynamic personality process rather than a personality trait [37, 46]. Therefore, resilience can have an effect as part of the sport efficiency of the athlete [47]. Resilient individuals exhibit behavioral autonomy by taking responsibility for their actions. They self-regulate by planning for and setting goals and then monitoring their progress toward these goals. The satisfaction of basic psychological needs, facilitated by supportive social contexts, appears both to foster a sense of wellness and to lead to building inner resources that underlie resilience [48].

### Hypotheses

**Fig 1:**
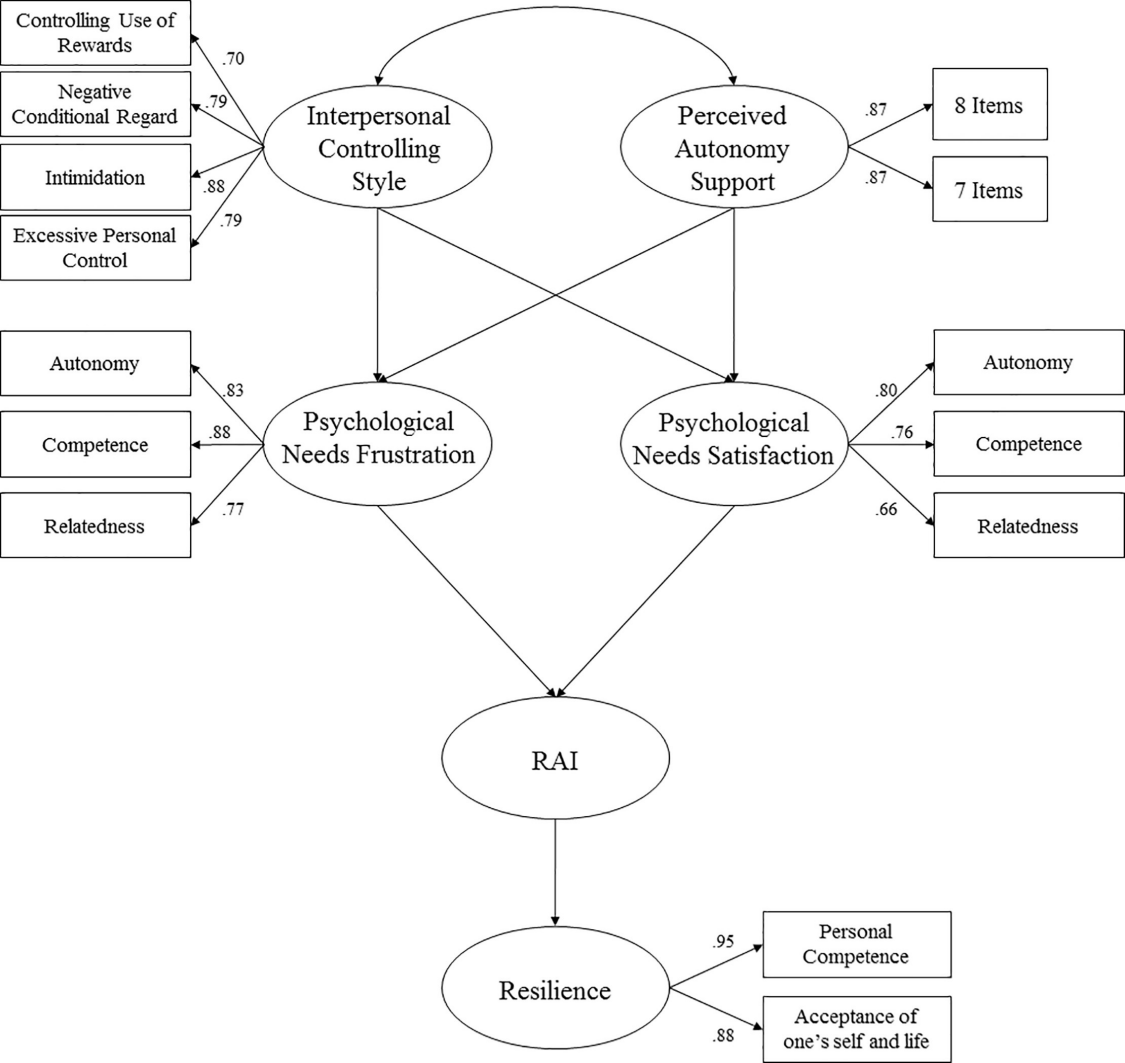
Hypothesized model in the sports context from the perspective of self-determination theory.

A consideration of previous studies led us to hypothesize that (Fig 1): (1) Perceived autonomy support by the trainer would positively affect the satisfaction of psychological needs and negatively affect the frustration of psychological needs; (2) a perceived controlling interpersonal style of the trainer would positively affect the frustration of psychological needs and negatively affect the satisfaction of psychological needs; (3) the satisfaction of basic psychological needs would positively affect autonomous motivation and negatively affect the frustration of psychological needs; and (4) autonomous motivation would affect resilience. In addition, we will examine the possible indirect effects of interpersonal trainer styles on motivations and resilience through the mediation of basic psychological needs.

## Method

### Design and participants

This cross-sectional study involved 324 sportspeople who were members of second-division sports clubs (161 male and 163 female) aged 18 to 34 years (*M* = 23.9; *SD* = 3.15). These athletes trained 4 days a week in addition to a weekly competition game.

### Procedure

In order to carry out this study, we contacted a variety of sports clubs in Spain. All participants were informed that they were to fill out a questionnaire and asked to provide written consent to participate. The questionnaire was administered anonymously under the supervision of an expert pollster, a member of the investigation group, who was able to provide explanations to resolve doubts that participants might have about the questionnaires. The Ethical Review Committee at the University of Almería approved the study.

### Measures

#### Perceived autonomy support

We used the Spanish Sport of Climate Questionnaire [7]. This instrument has its origin in the Health Care Climate Questionnaire (HCCQ, [49]) and comprises a total of 15 items in the expanded version. It evaluates the perception sportspeople have of the level of support of autonomy offered by their trainer. The answers were collected on a 7-point Likert scale ranging from *false* (1) to *very true* (7). In this study, Cronbach’s α for the global factor of autonomy support was .86.

#### The perception of the controlling interpersonal style of the coach

The Spanish version [50] of the Controlling Coach Behaviors Scale (CCBS, [15]), comprising 15 items divided into four subscales (controlling use of rewards, negative conditional attention, intimidation, excessive personal control), was used. The items were scored on a Likert scale. In this study, the value of Cronbach’s α was .86 for the global factor of interpersonal controlling style; in the four subscales, the values were: controlling use of rewards, .86; negative conditional attention, .88; intimidation, .90; and excessive personal control, .83.

#### The satisfaction of basic psychological needs in the sport context

This was measured with the *Escala de Mediadores Motivacionales en el Deporte* (EMMD; Motivational Mediators Scale in Sport) created by González-Cutre et al., 2007 [51]. This scale comprises items grouped in three factors: Seven items for perceived competence, eight items for autonomy, and eight items for relationship with others. The items were scored on a 7-point Likert scale ranging from 1 = *I totally disagree* to 7 = *I totally agree*. This scale has been widely used in several studies [52]. In this study, Cronbach’s α was .78 for the global factor of satisfaction; turning to the individual factors, satisfaction of autonomy had a score of .84, satisfaction of perceived competence was .81, and satisfaction with relatedness was .80.

#### The frustration of psychological needs in the sport contexts

This was evaluated through the Spanish version by Balaguer et al. [7] of the Psychological Needs Thwarting Scale (PNTS, [24]). This scale was composed of 12 items grouped in three subscales that assess the extent to which sportspeople perceive the frustration of their needs for competence, autonomy, and relatedness. The answers were collected on a 7-point Likert scale ranging from 1 = *I totally disagree* to 7 = *I totally agree*. In this study, Cronbach’s α was .88 for the global factor of thwarting, and took the following values for each subscale: Frustration competence, .88; frustration autonomy, .87; and frustration relatedness, .82.

#### Autonomous motivation

This was evaluated by means of the Behavioural Regulation in Sport Questionnaire of Lonsdale, Hodge, and Rose [53], validated and adapted to the Spanish context by Viladrich, Torregrosa, and Cruz [54], which was designed to evaluate the motivation for pursuing sports from the perspective of SDT. This scale was used to evaluate the reasons for participating in sport, with 24 items and six subscales that include intrinsic motivation, integrated regulation, identified regulation, introjected regulation, external regulation, and amotivation. Items were scored from 1 = *Absolutely false* to 7 = *Absolutely true*.

The relative autonomy index (RAI) was used to evaluate autonomous motivation. RAI was calculated as recommended by Vallerand [28] by assigning a weight to each type of motivation according to its place in the motivational continuum. The following formula was used to calculate the RAI: (3 × Intrinsic Mot.) + (2 × Integrated Reg.) + (1 × Identified Reg.) − (1 × Introjected Reg.) − (2 × External Reg.) − (3 × Amotivation).

#### Resilience

This factor was evaluated by means of the Scale of Resilience in the Sport Context (ERCD, [55]), adapted from the Portuguese version developed by Vigário, Serpa, and Rosado [56]. The scale comprises 25 items grouped into two factors, 17 of which correspond to personal competence and 8 to the acceptance of one’s self and life. The participants had to indicate their answer on a 7-point Likert scale ranging from 1 = *Disagree* to 7 = *Totally agree*. In this study, Cronbach’s α was .90 for the global factor of resilience.

### Data analysis

The predictive statistics were calculated first and a correlation analysis of the variables of the study conducted by means of Pearson’s correlation coefficients, together with an analysis of reliability through Cronbach’s α of every factor of the study.

Second, we examined the quality of the measurement models as a step preceding the SEM model estimation. For this, we estimated a confirmatory factor model that was congruent with the theoretical structure of the scales (six correlated factors) and an exploratory structural equation model (ESEM, Asparouhov & Muthén, 2009) [57] with six correlated factors. Since the assumption of conditional independence of the items in the independent clusters confirmatory model can be excessively restrictive [58], it is recommended that the CFA models be compared to their exploratory equivalents in order to determine if the restrictions imposed by the CFA (especially in the cross-loadings) are compatible with the reality of the data [59]. We used ESEM instead of EFA in light of such advantages of the former as its greater flexibility and the possibility of comparing it with nested confirmatory models using the same set of fit indices. For the estimation of the ESEM model, target rotation was used. This allows us to obtain a rotated solution that is closer to a predetermined matrix of primary and cross-loadings [60], enabling ESEM to operate in a semi-confirmatory mode [57].

To decide which measurement model to use in the SEM estimation, we compared the CFA and ESEM models. Major differences in fit and/or parameters suggest retaining the ESEM model; non-significant or irrelevant differences favor the CFA model, which is more parsimonious [59]. We compared the fit of the CFA and ESEM models, the congruence coefficients (rc) between the primary factor loadings (an rc of over .96 suggests that the compared factors may be considered equal) [61], the convergent validity of the factors via the average variance extracted (AVE; values above .50 suggest suitable convergent validity) [62], and the effect size of the cross-loadings over the uni-dimensionality of the item through the estimate of the common variance explained by the item (iECV). The iECV [63,64] estimates the degree to which the item responses are explained by the putative latent variable in contrast to the effects of other sources of systematic variance. In this study, we calculated the iECV, whereby the proportion of common variance explained in each item by the primary factor is estimated in contrast to the variance explained by the other factors through the cross-loadings. iECV values close to 1 suggest that the effect of the non-primary dimensions is weak, that there is not a relevant violation of conditional independence, that the item may be considered to be essentially uni-dimensional, and, in short, that specifying the cross-loadings as 0 in the CFA model is fundamentally correct.

Third, we carried out structural equation modeling (SEM) of the predicted relationships hypothesized in the model. When testing the effects of mediation among the different variables of the model, the premises established by Baron and Kenny [65] were taken into account: (a) Meaningful correlations between independent and dependent variables; (b) meaningful correlations between independent variables and mediators; (c) meaningful correlations between the mediators and dependent variables; and (d) the previous meaningful mediation between the independent and dependent variables stops being meaningful when relations between the independent variables and mediators and between the mediators and the dependent variables are controlled.

All models were estimated using robust maximum likelihood (MLR) implemented in Mplus 7.0 [66]. Despite the lack of normality of the hypothesized model (Mardia’s coefficient = 54.77), this procedure revealed the strength of the estimations [67]. To analyze the goodness of fit of the model, the following criteria were used: the coefficient *χ*^2^, chi-squared divided by degrees of freedom (*χ*^2^*/df*), the Comparative Fit Index (CFI), Tucker-Lewis Index (TLI), Root Mean Square Error of Approximation (RMSEA) plus its 90% confidence interval, and Standardized Root Mean Square Residual (SRMR). In general, acceptable results would be *χ*^2^*/df* below 5 [68], values of CFI and TLI equal to or greater than .95, values of .06 or less for RMSEA, and .08 or less for SRMR [68]. However, Marsh, Hau, and Wen (2004) [69] argue that these cut-off values are too restrictive and difficult to achieve when complex models are tested, so they should be interpreted with caution.

## Results

### Preliminary analysis

Table 1 presents the descriptive statistics and correlations among the different variables of the study. All the measures were moderate, although there were some with low values, in particular psychological-needs thwarting with (*M* = 2.10), while the highest was autonomous motivation (*M* = 13.15).

**Table 1:** Descriptive statistics and correlation analysis between the factors.

Factors	*M*	*DT*	1	2	3	3.1	3.2	3.3	4	4.1	4.2	4.3	5	6
**1. Controlling Coach**	2.15	.87		−.14**	.52**	.47**	.38**	.46**	−.27**	−.19**	−.27**	−.23**	−.44**	−.17**
**2. P. Autonomy Support**	4.76	1.28			−.24**	−.22**	−.22**	−.24**	.34**	.28**	.20**	.36**	.32**	.37**
**3. PNF**	2.10	1.06				.81**	.83**	.84**	−.35**	−.23**	−.37**	−.28**	−.53**	−.17**
**3.1. Autonomy Frustration**	2.02	1.10					.75**	.66**	−.29**	−.20**	−.28**	−.23**	−.50**	−.16**
**3.2. Competence Frustration**	2.24	1.24						.64**	−.34**	−.24**	−.35**	−.27**	−.45**	−.12*
**3.3. Relatedness Frustration**	2.05	1.25							−.30**	−.18**	−.34**	−.25**	−.47**	−.13*
**4. BPNS**	4.79	1.15								.84**	.80**	.85**	.44**	.54**
**4.1. Autonomy Satisfaction**	4.52	1.50									.50**	.61**	.44**	.46**
**4.2. Competence Satisfaction**	5.28	1.28										.55**	.44**	.37**
**4.3. Relatedness Satisfaction**	4.58	1.35											.32**	.42**
**5. RAI**	13.15	14.23												.58**
**6. Resilience**	4.59	1.67												

*p < .01

**p < .001.

Note: P = Perceived; PNF = Psychological Needs Frustration; BPNS = Satisfaction of Basic Psychological Needs; RAI = Relative Autonomy Index

Pearson’s correlation analysis shows how different the results are according to the postulates of SDT. We see that autonomy support, satisfaction, and RAI have a positive relation with resilience, while controlling style and thwarting have a negative relation with resilience.

### Measurement models

Table 2 shows the fit indices and parameters of the CFA and ESEM models (for the correct estimation of the ESEM model, it was necessary to constrain the residual variance of one item to be equal to or greater than zero). As expected, the ESEM model fitted better than the CFA model (S-B-scaled *χ*^2^ difference = 81.5 (36), *p* = .000; ΔRMSEA = −.015; ΔCFI = .024; ΔTLI = .020; ΔSRMR = −.026), suggesting that freeing up the cross-loadings led to a better specified measurement model. The factorial loadings of the CFA model were high (*M* = .81, *SD* = .08), with AVE values above .50 in all cases.

**Table 2:** Results of the CFA and ESEM models.

Parcel/factor	F1	F2	F3	F4	F5	F1	F2	F3	F4	F5	iECV
1	**.69**	0	0	0	0	**.67**	.10	−.14	.00	.01	.94
2	**.78**	0	0	0	0	**.76**	−.08	.04	.05	.01	.98
3	**.88**	0	0	0	0	**.87**	**−.09**	.03	.01	−.07	.98
4	**.78**	0	0	0	0	**.77**	.06	−.14	−.01	.03	.96
5	0	**.84**	0	0	0	.00	**.90**	.04	.01	−.02	.99
6	0	**.91**	0	0	0	−.01	**.81**	.00	−.03	.06	.99
7	0	0	**.75**	0	0	.03	.04	**.74**	.01	.05	.99
8	0	0	**.68**	0	0	−.04	−.05	**.61**	−.15	.05	.93
9	0	0	**.81**	0	0	−.02	.13	**.75**	.01	−.02	.97
10	0	0	0	**.87**	0	.05	.05	.05	**.86**	−.08	.98
11	0	0	0	**.85**	0	−.10	−.02	−.09	**.90**	.06	.98
12	0	0	0	**.76**	0	**.13**	−.08	.00	**.67**	.00	.95
13	0	0	0	0	**.96**	**−.04**	.01	−.01	.00	**.99**	.99
14	0	0	0	0	**.86**	.06	.01	.10	−.01	**.79**	.98
AVE	.61	.76	.56	.68	.83	.59	.73	.50	.66	.80	
McDonald’s ω	.86	.87	.80	.87	.90	.86	.86	.77	.85	.90	
Cronbach’s α	.86	.86	.78	.86	.90						
Model fit	RMSEA = .053; CFI = .969; TLI = .958; SRMR = .040.					RMSEA = .038; CFI = .993; TLI = .978; SRMR = .014.					

Note: AVE = Average Variance Extracted; iECV = item Explained Common Variance; in bold = significant loadings (*p* < .05); shaded cells = targeted loadings; RMSEA = root mean square error of approximation; CFI = comparative fit index; TLI = Tucker-Lewis index; SRMR = standardized root mean square residual.

The ESEM model recovered the theoretical structure of the scale with high precision, revealing high primary loadings (*M* = .79, *SD* = .10), as well as very small cross-loadings (*M* = .04, *SD* = .04) that were only significant in two cases (*p* < .05). The AVE values of the ESEM in all cases were higher than .50, suggesting an appropriate convergent validity of the factors. Collectively, the primary loadings explained more than 93% of the common variance, indicating that the cross-loadings had an irrelevant effect size. The primary loadings of the CFA and ESEM models were almost equivalent (rc = 1, .995, .999, .998, .997), suggesting an almost perfect congruence between the load patterns of both models. Finally, the iECV values were between .93 and .99, indicating a high uni-dimensionality of the items and a very weak effect of the cross-loadings. Given these results, we opted to use the CFA model, which is more parsimonious, as part of the SEM model.

### Structural equation modeling

Before testing the hypothesized model through SEM and analyzing the relationships between the variables in the model, a reduction in the number of latent variables was carried out, with each of these having at least two indicators [70]. Specifically, the frustration of basic psychological needs included three indicators (autonomy, competence, and relatedness, as suggested by Bartholomew et al., 2011) [24]; satisfaction of basic psychological needs (autonomy, competence, and relatedness, according to González-Cutre et al., 2007) [51]; trainer control (intimidation, excessive personal control, the controlling use of rewards, and negative conditional regard, as Bartholomew et al. suggested, 2010) [15]; and resilience (including personal competence and the acceptance of oneself and of life, as Vigário et al. suggested, 2009) [56]; and finally, in the support of autonomy, it was necessary to separate the 15 items of the scale into two indicators in order to identify the model, following McDonald and Ho (2002) [70]. Table 3 shows the direct, indirect, and total effects between the relationships of the variables.

**Table 3:** Direct, indirect, and total effect.

Variable	Direct Effect	Indirect Effect	Total Effect
Controlling style → Thwarting PN	.55***	.00	.55***
Controlling style → Satisfaction PN	−.27***	.00	−.27***
Autonomy Support → Thwarting PN	−.17***	.00	−.17***
Autonomy Support → Satisfaction PN	.39***	.00	.39***
Thwarting PN → RAI	−.46***	.00	−.46***
Satisfaction PN → RAI	.35***	.00	.35***
RAI → Resilience	.60***	.00	.60***
Controlling style → RAI (*through frustration of psychological needs*)	.00	−.20	−.20
Autonomy Support → RAI (*through satisfaction of psychological needs*)	.00	.35	.35
Controlling style → Resilience (*through frustration of psychological needs and RAI*)	.00	−.38*	−.38*
Autonomy Support → Resilience (*through satisfaction of psychological needs and RAI*)	.00	.16*	.16*
Thwarting PN → Resilience (*through RAI*)	.00	.21	.21
Satisfaction PN → Resilience (*through RAI*)	.00	.28	.28

*** p < .001

**p < .01

*p < .05

The hypothesized model of predictive relations (Fig 1) was tested, yielding acceptable goodness-of-fit indices: *χ*^2^(83, *N* = 324) = 278.44, *p* < .001; *χ*^2^*/df* = 3.35; CFI = .93; TLI = .91; IFI = .93; RMSEA = .08 (IC 90% = .074–.083); SRMR = .057.

*Hypothesis 1*. The trainer’s perceived interpersonal controlling style showed positive effects on the frustration of psychological needs (β = .55, *p* < .001) and negative effects regarding the satisfaction of basic psychological needs (β = −.27, *p* < .001).
*Hypothesis 2*. The perception of autonomy support showed positive effects of the satisfaction of the basic psychological necessities (β = .39, *p* < .001), as well as negative effects regarding the frustration of psychological needs (β = −.17, *p* < .01).
*Hypothesis 3*. The satisfaction of the basic psychological needs showed positive effects on autonomous motivation (β = .35, *p* < .001), but the frustration of psychological needs had negative effects on autonomous motivation (β = −.46, *p* < .001).
*Hypothesis 4*. Autonomous motivation had positive effects on the resilience (β = .60, *p* < .001).

The relation between the trainer’s interpersonal controlling style and the perception of autonomy support was negative (β = −.19, *p* < .01).

In addition, the relation between autonomy support and resilience through the mediators of need satisfaction and motivation was positive (β = .16, *p* < .05).

On the other hand, the relation between interpersonal controlling style and resilience through the mediators of need satisfaction and motivation was negative (β = −.38, *p* < .05).

## Discussion

The present study takes into account the dual role the trainer can adopt in relation to the satisfaction and frustration of the sportsperson’s basic psychological needs and motivation, as well as to resilience in the sports context of SDT. Most previous studies have analyzed the trainer’s support of autonomy in relation to the satisfaction of basic psychological needs [7,25]. However, in the past few years, other studies have taken into account the trainer’s interpersonal controlling style [71], including both interpersonal styles in relation to the frustration of basic psychological needs. Thus, this study seeks to examine this issue more deeply by applying SDT to analyze the influence of the trainer’s role on sportspeople’s psychological needs and resilience and their importance in the sportsperson’s social, emotional, and psychological development [72].

The results of this study have shown that the trainer’s interpersonal controlling style negatively affected the satisfaction of basic psychological needs and positively affected the frustration of psychological needs, and at the same time support to autonomy positively affected the satisfaction of basic psychological needs and negatively affected the frustration of psychological needs. The present study is in line with what was established by SDT, because if sportspeople perceive themselves as having a certain freedom and decision-making capacity, they will see their perceived competence, psychological well-being, and satisfaction of their basic psychological needs as favored; but if the trainers’ behavior is critical or restrictive, or even pressures sportspeople, they will feel oppressed, incapable, and rejected, perceiving an obstruction of their necessities [31,71,73,74].

The results also showed that the satisfaction of basic psychological needs positively affected autonomous motivation, while on the other hand the frustration of psychological needs negatively affected autonomous motivation. These results are very similar to those of previous studies, e.g., Balaguer et al. [7], Castillo et al. [50], and González-Cutre, Sicilia, Sierra, Ferriz, and Hagger [74], as feeling competent and capable during training and competitions, establishing good interpersonal relations between the team members with each other and/or the trainer, and feeling that they are owners of their own destiny help sportspeople to feel autonomous motivation for the sport they are practicing.

The results of this study suggest that autonomous motivation positively affected resilience. This can be understood from the fact that sportspeople with autonomous motivation will be able to face a series of adverse circumstances, potentially stressful ones, throughout their sports lives thanks to high and meaningful levels of effort, constancy, dedication, and sacrifice [1]. In addition, other studies have connected self-determined motivation with personal well-being at the emotional and cognitive levels [73].

Finally, we found that the indirect effect of the trainer’s interpersonal controlling style on resilience was negative, while the trainer’s support of autonomy affected resilience positively. Studies in the educational field confirm that autonomy positively affects resilience [72]; nevertheless, this is the first study to show a relationship between a trainer’s interpersonal style and a sportsperson’s resilience. This relation could be explained by the fact that sportspeople facing an adverse situation on their own undergo a learning process much deeper and more rewarding when they perceive autonomy support than when they are controlled by the trainer, apart from the fact that they will acquire certain abilities that are generalizable and translatable to other adverse situations [75].

The relationships between trainer and athlete are complex. This study seeks to raise awareness among trainers of the impact of their interpersonal styles on sportspeople’s psychological need satisfaction (vs. frustration), motivation, and resilience. Thus, coaches should not focus exclusively on the athlete’s technical, strategic, and tactical skills, but should also focus on developing effective relationships with their sportspeople [76]. Initially, the trainers may feel the need to be somewhat controlling and apply pressure to sportspeople so that they will put effort into the sport activity, but they need to realize that this can often damage sportspeople’s need satisfaction. Instead, striving to instruct sportspeople in autonomy-supportive ways, such as acknowledging their negative feelings toward certain tasks, providing them with specific rationales about why the basic skills are helpful for their sport training, and providing some choice when possible, will yield better results [14]. Finally, the autonomy support of coaches when developing effective relationships with their athletes could impact athletes’ resilience, resulting in improved performance and accomplishments.

Therefore, the prosocial skills of the trainer should be a very important element in future training courses, since, as shown in this study, the role adopted by the trainer would affect the performance and perception of athletes.

### Limitations and future directions of this research

This study applied the postulates of SDT to new variables to show its applicability to Spanish culture. However, after reviewing the findings of the model, it is necessary to emphasize that, as it is a correlational study, it does not allow us to infer cause and effect. The results we obtained could be interpreted differently depending on the approach one takes. The complexity of the model does not allow us to elaborate which type of motivation would be associated with resilience (i.e., whether type of motivation, such as intrinsic or integrated, and identified regulation, would be associated with positive adaptative behaviors). In addition, future studies should check how friends and family can influence the motivation and resilience of the athlete and which factors are more important until such time as the effects of these influences stabilize with further training. In addition, future studies could analyze longitudinally through experimental models how the coach’s controller role influences the resilience and performance of the athlete versus the role of supporting the coach’s autonomy. Also, we must not forget that as the study population was drawn from only one location, this study is not generalizable to the general population. As it seems likely that the model shows strong relations capable of generalization to different cultures and ages, the next step will be to perform an ethnographic study to understand the psychological aspects of sport in greater detail.

## Conclusion

In conclusion, this study provides support for the postulates of SDT. The results suggest a positive relation between a trainer’s autonomy support and the satisfaction of sportspeople’s basic psychological needs and resilience.

The coach’s perceived interpersonal controlling style is positively related with the thwarting of psychological needs and resilience. Autonomous motivation is related negatively to the thwarting and positively to the satisfaction of psychological needs, and finally, autonomous motivation predicts resilience.

## Supporting information

S1 File (SAV)
